# Supplementary material for: Individual Scent-Marks of Nest Entrances in the Solitary Bee, Osmia cornuta (Hymenoptera: Apoidea)
Source: Insects. 2021 Sep 18;12(9):843. doi: 10.3390/insects12090843 (PMC8466334; doi:10.3390/insects12090843)
Supplement: Supplementary file 1 [file insects-12-00843-s001.zip › insects-1376098-SI.pdf]

Supplementary Materials

Individual scent-marks of nest entrances in the solitary bee, *Osmia cornuta* (Hymenoptera: Apoidea)

Konrad Sebastian Frahnert and Karsten Seidelmann

Table S1. Substances identified in nest marks of *O. cornuta*, extracted by hexane from filter pater sleeves.

Peak	Tag	Name	Formula	Mol. Mass	RT[1] [min]	KI[2]
3	C17:0	n-Heptadecane	$C_{17}H_{36}$	240.47	7.230	1700
4	C15:0-Ald	Pentadecanal	$C_{15}H_{30}O$	226.40	7.373	1715
6	C14:0-EE	Tetradecanoic acid ethyl ester	$C_{16}H_{32}O_2$	256.42	8.055	1791
7	C16:0-Ald	Hexadecanal	$C_{16}H_{32}O$	240.43	8.328	1819
8	Met-C15-2-one	6.10.14-Trimethyl-pentadecan-2-one	$C_{18}H_{36}O$	268.28	8.481	1833
10	9-C19:1	(Z)-9-Nonadecene	$C_{19}H_{38}$	266.51	8.916	1875
11	7-C19:1	(Z)-7-Nonadecene	$C_{19}H_{38}$	266.51	8.980	1881
12	C19:0	n-Nonadecane	$C_{19}H_{40}$	268.52	9.171	1900
14	C17:0-Ald	Heptadecanal	$C_{17}H_{34}O$	254.45	9.408	1920
16	9-C16:1-EE	(Z)-9-Hexadecen acid ethyl ester	$C_{18}H_{34}O_2$	282.46	10.007	1970
17	C16:0-EE	Hexadecanoic acid ethyl ester	$C_{18}H_{36}O_2$	284.48	10.261	1992
18	9-C16:1-IPE	(Z)-9-Hexadecen acid isopropyl ester	$C_{19}H_{36}O_2$	296.49	10.347	1999
19	Met-HC 1	methyl-branched Hydrocarbon			10.467	2014
20	C16:0-IPE	Hexadecanoic acid isopropyl ester	$C_{19}H_{38}O_2$	398.50	10.578	2017
21	C18:0-Ald	Octadecanal	$C_{18}H_{36}O$	268.48	10.633	2021
22	9-C21:1	(Z)-9-Heneicosane	$C_{21}H_{42}$	294.56	11.292	2072
23	7-C21:1	(Z)-7-Hentricosene	$C_{21}H_{42}$	294.56	11.374	2079
24	9.12-C18:2-ME	Methyl (Z.Z)-9.12-octadecadienoate	$C_{19}H_{34}O_2$	294.48	11.544	2092
25	C21:0	n-Heneicosane	$C_{21}H_{44}$	296.57	11.641	2100
26	C21:0-One	Nonadecan-2-one	$C_{19}H_{38}O$	282.50	11.703	2104
27	C19:0-Ald	Nonadecanal	$C_{19}H_{38}O$	282.50	11.967	2123
28	9-C18:1-OOH	(Z)-9-octadecenoic acid	$C_{18}H_{34}O_2$	282.47	12.144	2136
29	9.12-C18:2-EE	Ethyl (9Z.12Z)-9.12-octadecadienoate	$C_{20}H_{36}O_2$	308.50	12.463	2159
30	C18:1-EE	Ethyl (Z)-9-octadecenoate	$C_{20}H_{38}O_2$	310.51	12.552	2165
31	C18:0-EE	Ethyl octadecenoate	$C_{20}H_{40}O_2$	312.53	12.907	2191
32	C22:0	n-Docosane	$C_{22}H_{46}$	310.60	13.017	2200
33	C20:0-Ald	Eicosanal	$C_{20}H_{40}O$	296.53	13.385	2225
35	9-C20:1-1-OH	(Z)-9-Eicosen-1-ol	$C_{20}H_{40}O$	296.53	13.988	2267
36	9-C23:1	(Z)-9-Tricosene	$C_{23}H_{46}$	322.61	14.076	2273
37	7-C23:1	(Z)-7-Tricosene	$C_{23}H_{46}$	322.61	14.171	2280
38	Met-HC 2	methyl-branched Hydrocarbon			14.310	2289
39	5-C23:1	(Z)-5-Tricosene	$C_{23}H_{46}$	322.61	14.310	2289
40	C23:0	n-Tricosane	$C_{23}H_{48}$	324.63	14.457	2300
41	C21:0-Ald	Heneicosanal	$C_{21}H_{42}O$	310.56	14.838	2326
42	C20:1-EE	Ethyl (Z)-9-icosenoate	$C_{22}H_{42}O_2$	338.57	15.371	2362
43	9-C24:1	(Z)-9-Tetracosene	$C_{24}H_{48}$	336.64	15.520	2373

Peak	Tag	Name	Formula	Mol. Mass	RT[1] [min]	KI[2]
44	7-C24:1	(Z)-7-Tetracosene	$C_{24}H_{48}$	336.64	15.629	2380
45	C24:0	n-Tetracosane	$C_{24}H_{50}$	338.65	15.910	2400
46	9.12-C18:2-BE	Butyl-9.12-octadecadienoate	$C_{22}H_{40}O_2$	336.55	16.082	2411
47	9.12.15-C18:3-BE	Butyl-9.12.15-octadecatrienoate	$C_{22}H_{38}O_2$	334.54	16.192	2419
48	C22:0-Ald	Docosanal	$C_{22}H_{44}O$	324.58	16.328	2428
49	9-C22:1-1-OH	9-Docosenol	$C_{22}H_{44}O$	324.58	16.898	2467
50	9-C25:1	(Z)-9-Pentacosene	$C_{25}H_{50}$	350.66	16.991	2474
51	7-C25:1	(Z)-7-Pentacosene	$C_{25}H_{50}$	350.66	17.136	2484
52	5-C25:1	(Z)-5-Pentacosene	$C_{25}H_{50}$	350.66	17.261	2492
53	C25:0	n-Pentacosane	$C_{25}H_{52}$	352.68	17.375	2500
54	9-C23:1-1-OH	(Z)-9-Tricosene-1-ol	$C_{23}H_{46}O$	338.61	18.419	2573
55	9-C26:1	(Z)-9-Hexacosene	$C_{26}H_{52}$	364.69	18.432	2574
56	7-C26:1	(Z)-7-Hexacosene	$C_{26}H_{52}$	364.69	18.542	2581
57	C26:0	n-Hexacosane	$C_{26}H_{54}$	366.71	18.809	2600
58	C24:0-Ald	Tetracosanal	$C_{24}H_{48}O$	352.64	19.266	2632
59	Met-HC 3	methyl-branched Hydrocarbon			19.680	2661
60	9-C24:1-1-OH	(Z)-9-Tetracosene-1-ol	$C_{24}H_{48}O$	352.64	19.809	2671
61	9-C27:1	(Z)-9-Heptacosene	$C_{27}H_{54}$	378.72	19.866	2675
62	7-C27:1	(Z)-7-Heptacosene	$C_{27}H_{54}$	378.72	19.979	2683
63	5-C27:1	(Z)-5-Heptacosene	$C_{27}H_{54}$	378.72	20.121	2693
64	C27:0	n-Heptacosane	$C_{27}H_{56}$	380.73	20.218	2700
65	Met-HC 4	methyl-branched Hydrocarbon			21.066	2760
66	Met-HC 5	methyl-branched Hydrocarbon			21.209	2770
67	7-C28:1	(Z)-7-Octacosene	$C_{28}H_{56}$	392.74	21.376	2783
68	C28:0	n-Octacosane	$C_{28}H_{58}$	394.76	21.609	2800
69	9-C26:1-1-OH	(Z)-9-Octacosen-1-ol	$C_{28}H_{56}O$	408.74	22.445	2862
70	9-C29:1	(Z)-9-Nonacosene	$C_{29}H_{58}$	406.77	22.618	2875
71	7-C29:1	(Z)-7-Nonacosene	$C_{29}H_{58}$	406.77	22.737	2884
72	5-C29:1	(Z)-5-Nonacosene	$C_{29}H_{58}$	406.77	22.866	2893
73	C29:0	n-Nonacosane	$C_{29}H_{60}$	408.79	22.950	2900
74	7-C31:1	(Z)-7-Hentricosene	$C_{31}H_{62}$	434.82	25.354	3085
75	C31:0	n-Hentricosane	$C_{31}H_{64}$	436.84	25.561	3100

[1] RT = Retention Time
[2] KI = Kovats-Index

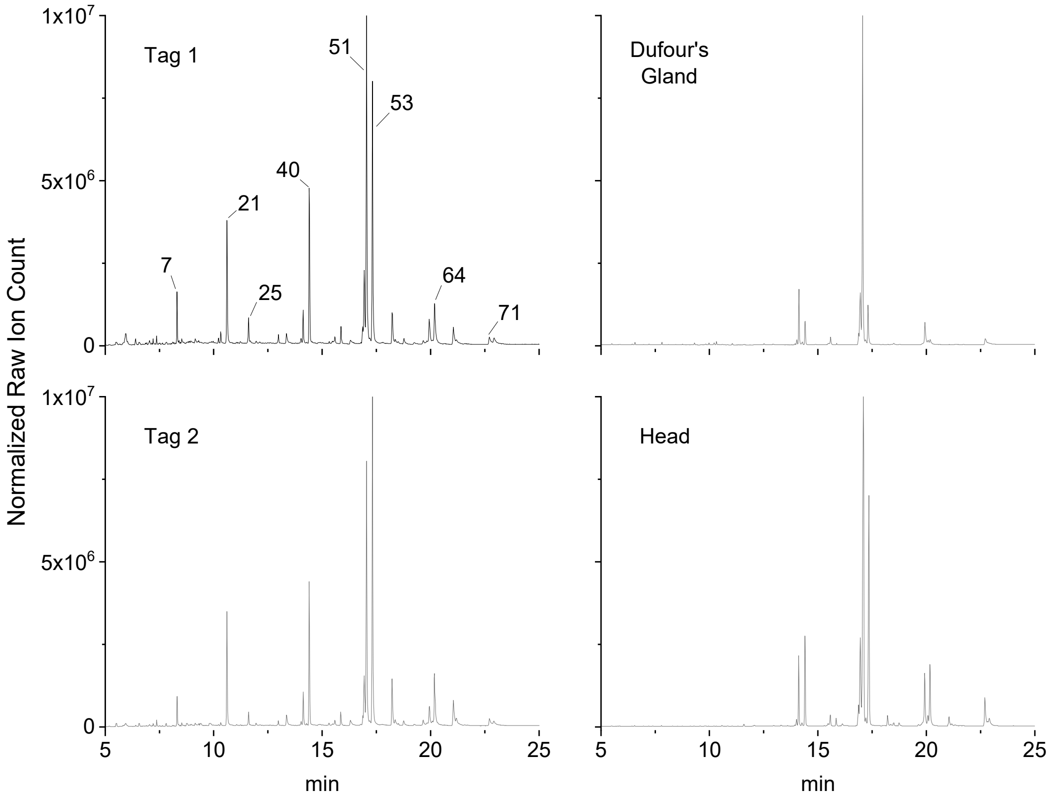

Figure S1. Chromatograms of the four-sample set from O. cornuta female C11. Chromatograms were normalized to the largest peak. For better orientation, some of the major compounds were marked for Tag 1. Numbers refer to substances in Table S1.

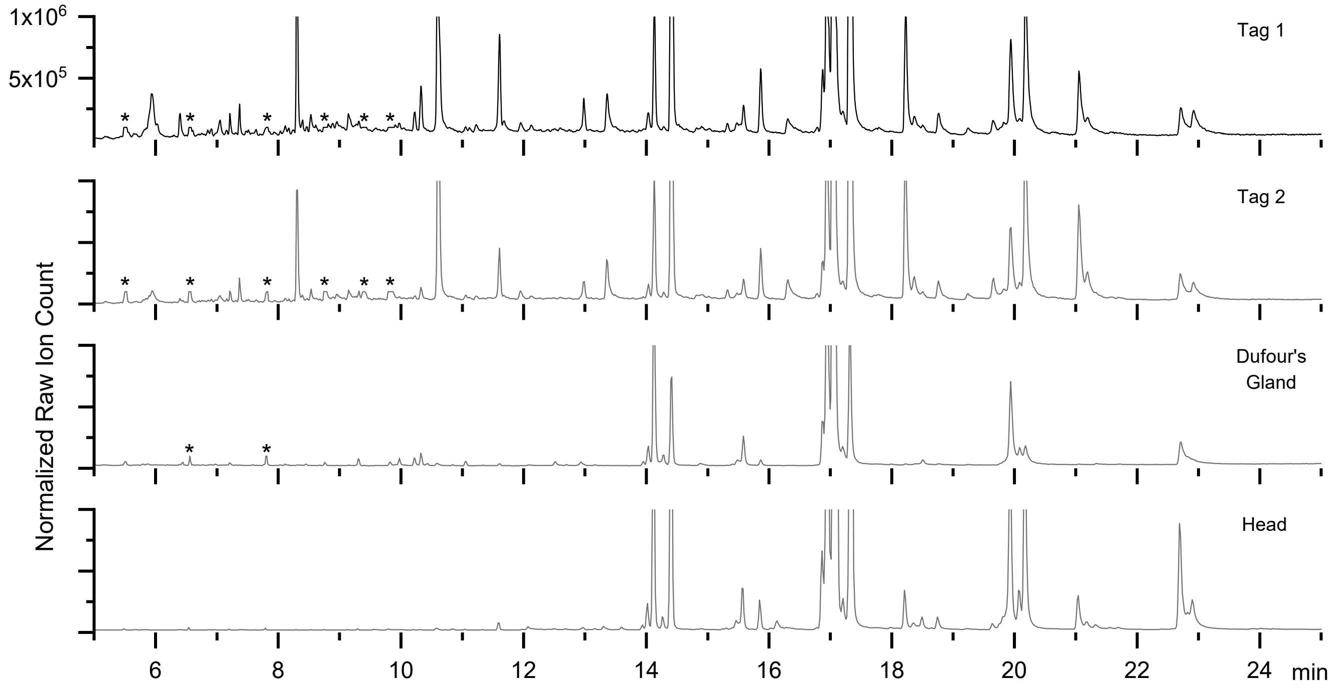

Figure S2. The juxtaposition of the normalized chromatograms of the four-sample set from O. cornuta female C11 (see Figure S1). Chromatograms were scaled to 1×10^6 to also show smaller compounds. Asterisks indicate matrix compounds that were clipped of for clarity of presentation.

Table S2. Quantity of substances found in different origins of volatiles. Only substances detectable in a sufficient number of samples were considered. The sum of all peak areas was normalized to 1×10^7 for each sample prior to calculating the mean and standard deviation. As in the tube, only a fraction of substances were found, and the sample peak sum was normalized to 4×10^5 to retain comparability (estimated by peaks originating from the solvent and GC column).

ID	Tag	Head		Gland		Nest Mark		Tube	
		nq[1]	mean ± sd	nq[1]	mean ± sd	nq[1]	mean ± sd	nq[1]	mean ± sd
3	C17:0	0	1.412 ± 312	0	13.133 ± 13.591	0	17.321 ± 5.266	3	
4	C15:0-Ald	25		25		0	16.325 ± 7.697	3	
6	C14:0-EE	25		0	5.214 ± 2.801	0	915 ± 1.003	3	
7	C16:0-Ald	0	816 ± 366	0	3.574 ± 5.164	0	123.558 ± 62.293	0	578 ± 243
8	Met-C15-2-one	23	61 ± 236	0	5.333 ± 2.820	38	432 ± 794	3	
10	9-C19:1	0	206 ± 380	23	218 ± 947	0	1.833 ± 3.125	0	125 ± 95
11	7-C19:1	0	136 ± 96	17	880 ± 1.546	0	4.476 ± 2.700	0	1.166 ± 400
12	C19:0	0	2.795 ± 2.372	0	4.731 ± 5.467	0	25.214 ± 30.591	0	687 ± 235
14	C17:0-Ald	12	230 ± 298	25		0	32.670 ± 17.027	2	86 ± 149
16	9-C16:1-EE	25		0	13.497 ± 6.202	20	926 ± 1.027	3	
17	C16:0-EE	25		0	4.781 ± 3.534	0	915 ± 1.108	3	
18	9-C16:1-IPE	11	403 ± 517	0	8.306 ± 9.979	0	15.391 ± 11.146	3	
19	Met-HC 1	25		0	6.897 ± 3.559	51	6 ± 47	3	
20	C16:0-IPE	20	390 ± 1.293	0	6.706 ± 6.471	0	15.921 ± 9.595	0	3.014 ± 742
21	C18:0-Ald	0	2.878 ± 1.197	10	5.440 ± 6.132	0	380.533 ± 190.228	3	
22	9-C21:1	0	496 ± 1.126	15	513 ± 1.046	4	1.477 ± 1.637	0	223 ± 69
23	7-C21:1	0	995 ± 369	0	3.195 ± 1.841	0	1.423 ± 1.101	0	253 ± 297
24	9,12-C18:2-ME	8	149 ± 132	25		25	848 ± 1.995	3	
25	C21:0	0	36.998 ± 36.960	0	21.618 ± 14.922	0	118.923 ± 60.217	0	2.515 ± 450
26	C21:0-One	0	2.237 ± 9.860	19	356 ± 1.057	0	11.133 ± 28.153	3	
27	C19:0-Ald	25		25		0	7.867 ± 3.114	3	
28	9-C18:1-OOH	0	15.860 ± 14.910	0	4.325 ± 4.895	0	1.226 ± 999	3	
29	9,12-C18:2-EE	25		24	453 ± 2.266	51	33 ± 236	3	
30	C18:1-EE	25		0	10.578 ± 5.681	13	1.308 ± 1.660	3	
31	C18:0-EE	14	34 ± 44	0	1.831 ± 1.611	0	711 ± 913	3	
32	C22:0	0	5.814 ± 3.292	0	5.869 ± 2.126	0	23.813 ± 7.225	0	2.025 ± 454
33	C20:0-Ald	10	1.444 ± 1.497	25		0	61.003 ± 26.077	3	
35	9-C20:1-1-OH	0	3.899 ± 1.407	0	9.505 ± 4.171	0	2.389 ± 1.130	3	
36	9-C23:1	0	38.294 ± 7.824	0	71.698 ± 21.349	0	32.699 ± 39.995	3	
37	7-C23:1	0	441.519 ± 131.923	0	831.316 ± 299.661	0	240.790 ± 99.852	3	
38	Met-HC 2	25		0	7.202 ± 3.641	46	165 ± 512	3	
39	5-C23:1	0	16.720 ± 5.087	0	35.648 ± 12.913	0	10.279 ± 5.067	3	
40	C23:0	0	577.822 ± 122.651	0	478.860 ± 157.263	0	790.774 ± 125.127	0	3.830 ± 108
41	C21:0-Ald	24	44 ± 220	25		0	7.012 ± 4.318	3	
42	C20:1-EE	25		24	232 ± 1.160	50	39 ± 216	3	
43	9-C24:1	0	15.291 ± 3.671	0	21.724 ± 7.677	0	11.428 ± 4.640	3	
44	7-C24:1	0	83.283 ± 11.756	0	130.965 ± 23.635	0	49.135 ± 17.290	3	
45	C24:0	0	63.609 ± 6.611	0	44.177 ± 11.149	0	79.978 ± 9.318	0	3.470 ± 326
46	9,12-C18:2-BE	0	1.346 ± 1.488	24	11 ± 53	46	32 ± 115	3	
47	9,12,15-C18:3-BE	0	8.030 ± 9.967	25		52		3	
48	C22:0-Ald	7	2.233 ± 2.058	23	349 ± 1.250	0	52.603 ± 24.112	2	92 ± 159
49	9-C22:1-1-OH	0	52.041 ± 28.238	0	92.033 ± 44.886	0	40.945 ± 28.189	3	
50	9-C25:1	0	536.599 ± 112.707	0	681.634 ± 149.345	0	333.820 ± 130.569	2	122 ± 211
51	7-C25:1	0	3.632.199 ± 355.500	0	4.589.903 ± 546.294	0	1.915.463 ± 614.592	0	1.189 ± 443

ID	Tag	Head		Gland		Nest Mark		Tube	
		nq[1]	mean ± sd	nq[1]	mean ± sd	nq[1]	mean ± sd	nq[1]	mean ± sd
52	5-C25:1	0	30.713 ± 3.668	0	41.948 ± 10.273	0	17.635 ± 7.853	3	
53	C25:0	0	2.040.344 ± 249.085	0	979.304 ± 270.559	0	1.767.645 ± 324.140	0	5.390 ± 2.051
54	9-C23:1-1-OH	0	66.426 ± 40.419	0	9.716 ± 10.620	0	169.883 ± 78.484	0	388 ± 259
55	9-C26:1	0	7.587 ± 2.646	0	6.513 ± 3.100	0	8.468 ± 4.057	3	
56	7-C26:1	0	32.411 ± 6.003	0	36.449 ± 8.615	0	17.794 ± 7.627	3	
57	C26:0	0	41.654 ± 6.157	0	16.315 ± 6.266	0	40.464 ± 8.148	0	1.961 ± 899
58	C24:0-Ald	10	1.524 ± 1.715	23	1.775 ± 6.672	0	24.612 ± 14.522	3	
59	Met-HC 3	0	10.418 ± 8.376	22	533 ± 1.724	0	22.973 ± 13.410	2	104 ± 180
60	9-C24:1-1-OH	0	7.874 ± 4.232	0	7.296 ± 4.147	0	5.423 ± 4.876	3	
61	9-C27:1	0	31.759 ± 16.801	0	49.587 ± 75.830	0	29.217 ± 25.942	3	
62	7-C27:1	0	476.430 ± 65.716	0	511.565 ± 76.002	0	247.167 ± 92.823	3	
63	5-C27:1	0	82.901 ± 16.911	0	78.944 ± 24.136	0	34.619 ± 18.162	3	
64	C27:0	0	743.772 ± 97.150	0	219.833 ± 80.842	0	502.110 ± 120.199	0	2.560 ± 1.191
65	Met-HC 4	0	80.450 ± 51.478	0	7.329 ± 9.325	0	150.556 ± 80.616	0	516 ± 327
66	Met-HC 5	0	16.794 ± 13.047	13	1.146 ± 1.893	0	25.833 ± 19.110	3	
67	7-C28:1	0	17.951 ± 4.604	0	18.410 ± 6.005	0	8.245 ± 4.643	3	
68	C28:0	0	9.803 ± 2.925	0	2.691 ± 1.597	0	8.596 ± 5.250	0	767 ± 299
69	9-C26:1-1-OH	11	1.794 ± 3.716	25		0	3.178 ± 3.322	2	47 ± 81
70	9-C29:1	0	2.501 ± 1.408	10	660 ± 1.075	0	3.918 ± 4.153	2	68 ± 118
71	7-C29:1	0	387.498 ± 68.853	0	333.176 ± 105.781	0	177.633 ± 81.385	3	
72	5-C29:1	0	48.179 ± 16.946	0	53.671 ± 24.299	0	17.507 ± 8.364	3	
73	C29:0	0	132.169 ± 28.558	0	18.640 ± 12.906	0	87.696 ± 48.464	0	17.589 ± 10.793
74	7-C31:1	0	110.075 ± 50.977	0	44.956 ± 27.486	0	30.524 ± 20.476	3	
75	C31:0	0	35.922 ± 11.300	14	2.465 ± 3.229	0	15.860 ± 9.072	0	1.028 ± 551

[1] nq = number of samples where substance was not quantifiable

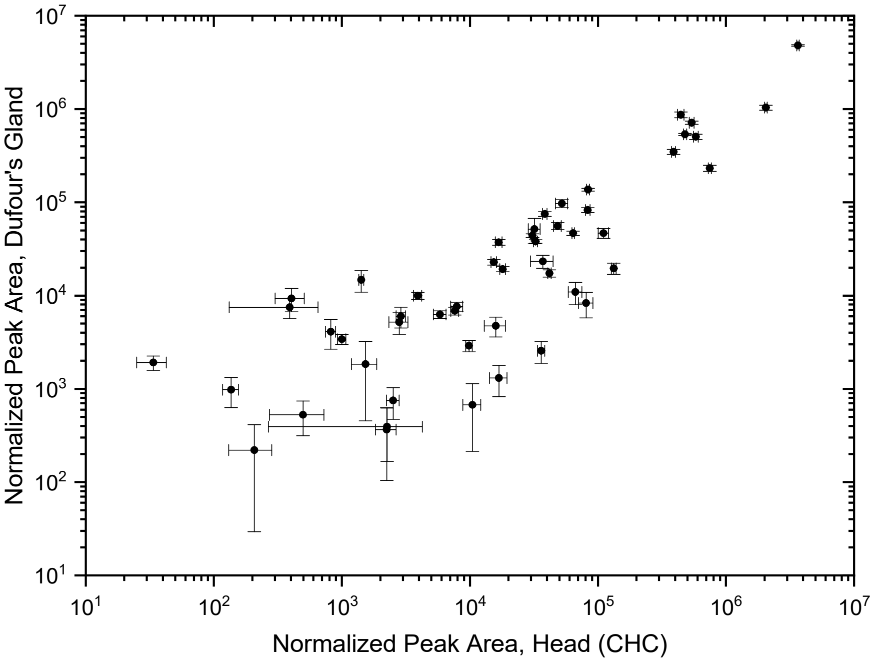

Figure S3. Correlation of the mean amounts ± sd (based on the largest diagnostic fragment) of 51 substances found both in Dufour's gland and in head extracts of *O. cornuta* females. Please note the logarithmic scale of axes.
